# *Bacteroidales*-Specific Antimicrobial Genes Can Influence the Selection of the Dominant Fecal Strain of *Bacteroides vulgatus* and *Bacteroides uniformis* from the Gastrointestinal Tract Microbial Community

**DOI:** 10.3390/life14050555

**Published:** 2024-04-26

**Authors:** Hyunmin Koo, Casey D. Morrow

**Affiliations:** 1 Department of Genetics, Hugh Kaul Precision Medicine Institute, University of Alabama at Birmingham, Birmingham, AL 35294, USA; 2 Department of Cell, Developmental and Integrative Biology, Hugh Kaul Precision Medicine Institute, University of Alabama at Birmingham, Birmingham, AL 35294, USA

**Keywords:** *Bacteroidales*-specific antimicrobial genes, antibiotics, microbial community, fecal microbial strain

## Abstract

*Bacteroides vulgatus* and *Bacteroides uniformis* are known to be abundant in the human fecal microbial community. Although these strains typically remain stable over time in humans, disruption of this microbial community following antibiotics resulted in the transient change to new strains suggesting that a complex, dynamic strain community exists in humans. To further study the selection of dominant fecal microbial strains from the gastrointestinal tract (GIT) community, we analyzed three longitudinal metagenomic sequencing data sets using BLAST+ to identify genes encoding *Bacteroidales*-specific antimicrobial proteins (BSAP) that have known functions to restrict species-specific replication of *B. uniformis* (BSAP-2) or *B. vulgatus* (BSAP-3) and have been postulated to provide a competitive advantage in microbial communities. In the HMP (Human Microbiome Project) data set, we found fecal samples from individuals had *B. vulgatus* or *B. uniformis* with either complete or deleted BSAP genes that did not change over time. We also examined fecal samples from two separate longitudinal data sets of individuals who had been given either single or multiple antibiotics. The BSAP gene pattern from most individuals given either single or multiple antibiotics recovered to be the same as the pre-antibiotic strain. However, in a few individuals, we found incomplete BSAP-3 genes at early times during the recovery that were replaced by *B. vulgatus* with the complete BSAP-3 gene, consistent with the function of the BSAP to specifically restrict *Bacteroides* spp. The results of these studies provide insights into the fluxes that occur in the *Bacteroides* spp. GIT community following perturbation and the dynamics of the selection of a dominant fecal strain of *Bacteroides* spp.

## 1. Introduction

Numerous longitudinal studies have used metagenomic sequencing coupled with new informatics approaches to establish the dominant fecal microbial community at the strain level is unique to the individual and generally stable over time [1,2,3,4]. However, the stability of dominant fecal microbial strains can be influenced by gastrointestinal tract (GIT) perturbations, such as antibiotics, that result in the transient appearance of new strains, although this can vary between individuals [5,6,7]. Usually after a short time, the new fecal strains are replaced with the previous dominant strains supporting the existence of a dynamic GIT ecosystem with multiple microbial strains that compete for fecal dominance [7].

In a recent study, Roodgar et al. showed that microbial strains found in the feces were probably stably maintained in GIT reservoirs defined by metabolic or spatial niches [8]. How these specific strains are maintained in these niches is unknown. The *Bacteroides vulgatus* and *Bacteroides uniformis* are known to be abundant in the human fecal microbial community [1,9,10]. We have previously shown that dominant fecal *B. vulgatus* or *B. uniformis* strains are unique to the individual and related to each other as determined from longitudinal strain tracking analysis [2,4,7,11,12]. Microbes are known to encode a variety of proteins that have the potential to provide a competitive advantage in a complex ecosystem [13,14,15,16]. Previous studies have identified the *Bacteroidales*-specific antimicrobial proteins (BSAPs) that have known functions to restrict species-specific replication and colonization of *B. uniformis* (BSAP-2) and *B. vulgatus* (BSAP-3) [9,15]. BSAPs have been shown to encode membrane attack complex/perforin (MACPF) signatures in proteins that lyse cells by pore formation and target the predominant LPS specific for *B. uniformis* (BSAP-2) or *B. vulgatus* (BSAP-3) [17]. The presence of these microbial genes in an individual’s *Bacteroides* spp. might then provide a competitive advantage to maintain that strain in the GIT ecosystem [14].

Based on the known functions of the BSAP genes, the objective of our study is to assess a possible role in the selection of the fecal dominant strain. We have determined the status of these genes in *B. vulgatus* or *B. uniformis* in fecal samples from studies that contained longitudinal metagenomic data sets from normal and antibiotic-treated individuals. The Integrative Genomics Viewer (IGV) has been used to visualize the genomic region (5′ and 3′ sequences) containing BSAP genes to determine the status of the BSAP genes in the individuals. Our study establishes the existence and long-term stability of the BSAP gene in individuals. Additionally, analysis of the data set from two studies using different antibiotics suggests a role for the BSAP genes in the selection of dominant fecal strains following the disruption of the GIT microbial ecosystem.

## 2. Materials and Methods

### 2.1. Publicly Available Data Sets Were Used in This Study

In this study, we used 3 publicly available metagenomic data sets for healthy individuals (1) from the NIH Human Microbiome Project (HMP, https://portal.hmpdacc.org/, accessed on 13 June 2012) [17], (2) pre and post treated with a single antibiotic (cefprozil) from Raymond et al. [18] (accession number: PRJEB8094), and (3) pre and post treated with 3 antibiotics (meropenem, gentamicin, and vancomycin) from Palleja et al. (accession number: ERP022986) [19]. The sequence data that we used in this study was fully anonymized before being made publicly available. For all three studies, longitudinal fecal samples were all taken from normal individuals with no known diseases. Further information on the characteristics of the individuals can be found in the primary references. For the HMP data set, 30 individual samples that were previously used to establish our window-based single-nucleotide variant (SNV) similarity (WSS) analysis were selected to run the analysis [2,17]. For Raymond et al., fecal samples were collected from 18 individuals at 3 different time points: pre-treatment (Day 0), end of antibiotic (cefprozil) treatment (Day 7), and 3 months post-treatment (Day 90), and we selected all individuals’ samples to run the analysis. We have also additionally selected 6 individual fecal samples that did not receive antibiotic treatment as controls. For the Palleja data set, fecal samples from 12 individuals treated with 3 antibiotics (meropenem, gentamicin, and vancomycin) were collected at 5 different time points: pre-treatment (Day 0), immediately after antibiotics treatment (Day 4), and 3 post-treatment time points (Day 8, 42, and 180). From this data set, we have selected all 12 individual samples at 5 different time points (pre and post treatment (Day 4, 8, 42, and 180) to run the analysis. 

### 2.2. Analysis of Bacteroides BSAP Genes

Before running the analysis, quality control steps included removing any human reference genome (hg19) using bowtie2 (version 2.3.4.3) with default parameters [20], and filtering low-quality reads (sliding window of 50 bases having a QScore < 20) using Trimmomatic (version 0.36) [21]. Each fecal metagenomics sample was used to align with *Bacteroides vulgatus* CL09T03C04 for BSAP-3 and *Bacteroides uniformis* CL03T00C23 for BSAP-2 using the Burrows–Wheeler aligner program (BWA; version 0.7.13) BWA tool [22]. Aligned reads from each reference genome were then sorted and indexed using SAMtools (version 0.1.19) [23]. The resultant bam file was converted to FASTQ format using BEDTools (version 2.26.0) [24]. Each converted FASTQ file was then assembled using MEGAHIT, and the resultant contig file was selected for BLASTX search against the BSAP-2 and BSAP-3 sequence reads using BLAST+ [15,25]. To visualize aligned Bacteriocin genes (BSAP-2 and BSAP-3) for paired samples for each individual, the VCF file generated for *B. vulgatus* and *B. uniformis* of each sample was uploaded to the Integrative Genomics Viewer (IGV) and aligned to their reference genome [16]. For the BSAP-3, the region of nucleotides located between nucleotides 2,178,016 to 2,183,973 of *B. vulgatus* was selected to display 5′ and 3′ sequences that also include BSAP-3 genes. For the BSAP-2, nucleotide regions of *B. uniformis* were selected to show 5′ and 3′ sequences that also include BSAP-2 genes (location: 1,383,872–1,385,398). To further characterize the BSAP negative phenotype, nucleotide sequences of the selected 5′ and 3′ regions were extracted using IGV and then uploaded to Jalview for visualization [16,26].

### 2.3. Analysis of Bacteroides *spp.* Strain Relatedness Using WSS

To determine the relatedness of strains, individual paired samples were additionally used for the Window-based single-nucleotide variant (SNV) similarity (WSS) analysis which was previously developed based on the Human Microbiome Project (HMP) data set [2]. The resultant WSS score was used to compare against the cut-off value that was previously established in our previous study (related strain pair: WSS score > cut-off; unrelated strain pair: WSS score < cut-off) (Supplementary Material) [2,27,28].

## 3. Results

Previous studies have reported the identification of the BSAP genes that are specific for *B. vulgatus* (BSAP-3) or *B. uniformis* (BSAP-2) [13,29]. For BSAP-3, protein signatures identifying MACPF that had activity to restrict growth in a species-specific manner of *B. vulgatus* were identified in gene cluster 16 from B. vulgatus/B. dorei. Similarly, in *B. uniformis*, MACPF was also identified as a protein encoded in gene cluster 15 that could restrict species-specific replication of *B. uniformis,* which was named BSAP-2. To determine the presence of the gene encoding BSAP-3 or BSAP-2, the individual samples from three longitudinal data sets used in this study were used for the gene analysis of clusters 15 and 16 using BLAST+ [15] and IGV tools [16].

**Figure 1:**
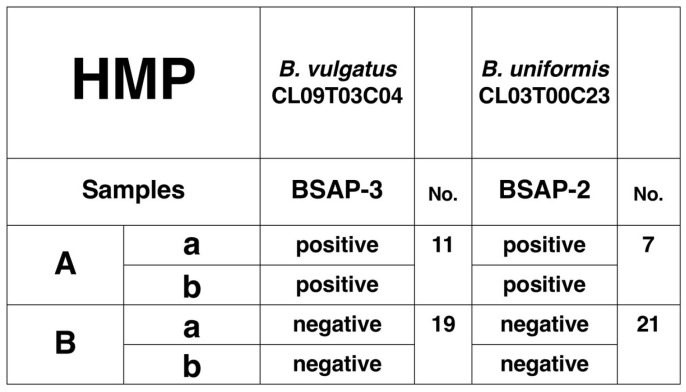
BSAP summary for HMP data set. The number of individual pairs that (A) both pairs were BSAP-2 and BSAP-3 complete (denoted as positive) and (B) both pairs were BSAP-2 and BSAP-3 incomplete (denoted as negative).

We first analyzed the human microbiome (HMP) data set that is composed of a longitudinal study of paired samples taken at separate times (up to 1 year apart) [1,17]. We selected 30 paired samples that we have previously shown to have related strains of *B. vulgatus* and *B. uniformis* over periods between 3 months and 1 year [2]. We screened for the complete genes encoding BSAP-3 for *B. vulgatus* and BSAP-2 for *B. uniformis* in each sample pair (Supplementary Table S1). We identified 11 sample pairs out of 30 for the complete gene encoding BSAP-3 and 7 of 28 samples for the gene encoding BSAP-2 (2 sample pairs, S64 and S70, had WSS scores below the cutoff value for *B. uniformis*) (Figure 1). For 30 BSAP-3 and 28 BSAP-2 sample pairs, we found the same BSAP (either positive or negative) for time points taken at times that were different by 3–6 months.

**Figure 2:**
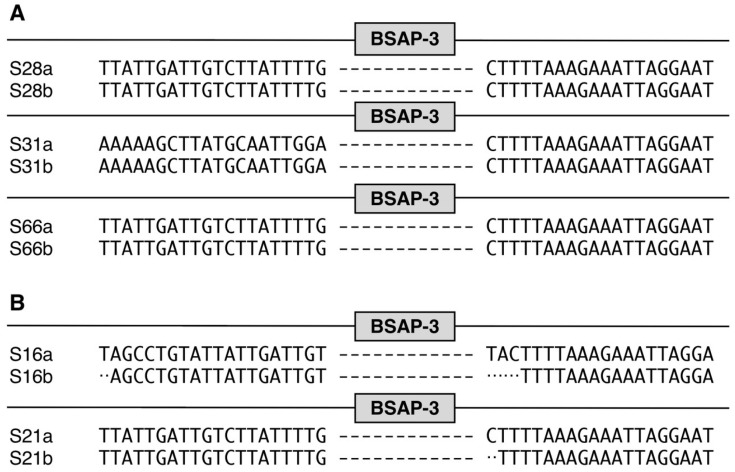
BSAP-3 gene alignment for HMP data set. Individual pairs that both pairs had BSAP-3 negative were selected to extract aligned sequence reads that included the BSAP-3 gene along with 5′ and 3′ of the BSAP-3 gene (20 base pairs for each 5′ and 3′ end). (**A**) Both individual pairs had exacted 5′ and 3′ sequence reads; (**B**) minor differences (less than 5 nucleotides) were observed between individual pairs; Major differences (more than 5 nucleotides) were detected between individual pairs.

**Figure 3:**
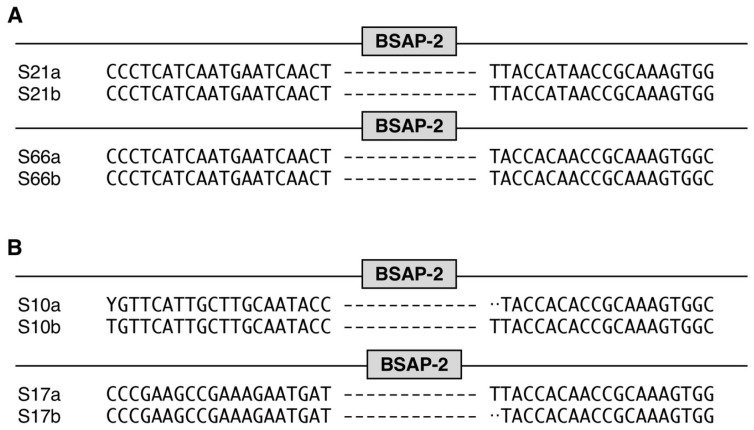
BSAP-2 gene alignment for HMP data set. Individual pairs that both pairs had deleted BSAP-2 genes were selected to extract aligned sequence reads that included the BSAP-2 gene along with 5′ and 3′ of the BSAP-2 gene (20 base pairs for each 5′ and 3′ end). (**A**) Both individual pairs had exacted 5′ and 3′ sequence reads; (**B**) minor differences (less than 5 nucleotides) were observed between individual pairs; Major differences (more than 5 nucleotides) were detected between individual pairs. “Y” and “S” are degenerate nucleotide code: “Y” indicates C/T and “S” indicates C/G.

To further characterize the BSAP genes that were incomplete (i.e., negative), we used the IGV to display the 5′ and 3′ sequences bracketing the BSAP deletion [16] (Supplementary Figure S1A,B). We found that 4 of the 19 sample pairs for the deleted BSAP-3 gene were identical (S28, S31, S48, and S66) and 10 of the 19 sample pairs (S16, S17, S21, S23, S49, S60, S64, S65, S69, and S70) had a few nucleotide differences bracketing the deletion from the comparison of the two (a and b) time points (Figure 2A,B and Figure S1A). Similarly, for BSAP-2 from the HMP, we found 2 of the 20 (S21 and S66) with deleted BSAP-2 had identical 5′ and 3′ sequences and 18 of the 20 sample pairs (S5, S10, S14, S16, S17, S19, S20, S23, S24, S28, S29, S31, S48, S51, S57, S60, S61, and S65) had only a few nucleotide differences in 5′ and 3′ sequences bracketing the deletion from the comparison of the a and b time points (Figure 3A,B and Figure S1B).

From a further inspection of the BSAP negative phenotype sample sets with IGV, we found a subset of individuals for BSAP-3 (S5, S14, S18, S62, and S63) and one individual (S49) for BSAP-2 that lacked a complete BSAP gene but did have a gene sequence fragment in the BSAP gene region. The BSAP-3 in individual S14 was particularly interesting since the *B. vulgatus* in the two samples were related as determined by WSS, with the first sample containing a deleted BSAP-3 while the second, later sample had an incomplete BSAP-3 gene (Supplementary Figure S1A). We also found that the pattern of the incomplete BSAP genes in these individuals was unique to that sample and thus differed between the two sample times. Thus, these results establish for both *B. vulgatus* and *B. unformis* that the BSAP patterns were generally stable over an extended time (3–6 months) although we did find some individuals with incomplete BSAP genes that had a few nucleotide differences between the two time points.

We next wanted to determine the BSAP pattern following perturbation and recovery of the gut ecosystem. For this analysis, we analyzed a data set from a previous study that analyzed gut microbes following a single antibiotic treatment [7,18]. The Raymond et al., data set contained the collection of fecal samples from six individuals at day 0, day 7 and day 90 that were used as untreated controls (Supplemental Table S2). Analysis of the BSAP genes from these samples found that three of the six had complete BSAP-3 genes (i.e., positive), while two of the six were positive for BSAP-2 (Supplementary Table S2 and Supplementary Figure S2A,B). The analysis of the samples from individuals with no antibiotics in the Raymond et al. study gave similar results as that found for the analysis of the HMP data set (Supplementary Table S2 and Supplementary Figure S2A,B).

**Figure 4:**
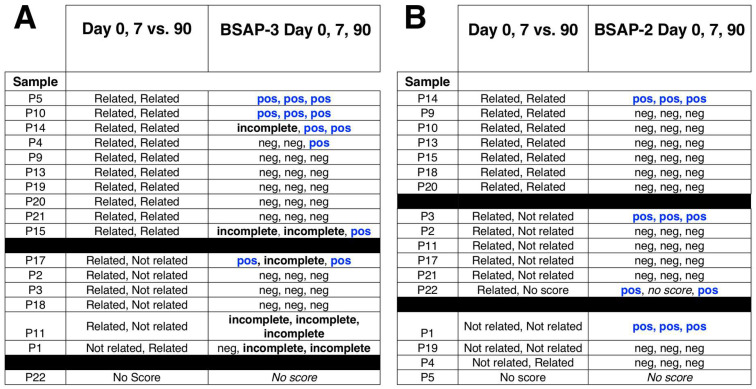
BSAP summary table for Raymond et al. data set. Gene analysis that includes WSS and BLAST was conducted for the Raymond et al. data set. For each sample, strain relatedness was determined by WSS analysis, and the presence/absence of (**A**) BSAP-3 and (**B**) BSAP-2 genes were determined by BLAST analysis. The “pos” indicates the complete BSAP-3/BSAP-2 gene was observed; “incomplete” indicates a sequence fragment of BSAP-3/BSAP-2 gene was observed; “neg” indicates BSAP-3/BSAP-2 gene was not observed; “No score” indicates that no score was observed from WSS analysis.

We next selected 17 individual sample sets from the Raymond et al. study that were given cephalexin. The samples were collected one day after treatment and 82 days after treatment (a total of 90 days after the pre-sample). We had previously analyzed this data set using our strain tracking analysis to show recovery of the pre-antibiotic strain at the day 90 samples for most individuals. Analysis of BSAP genes from the pre-sample demonstrated that 3 out of 17 of the BSAP-3 and 4 out of 17 of the BSAP-2 were complete (Figure 4). In most of the sample sets the BSAP phenotype did not change following antibiotics. We did find an incomplete BSAP-3 gene in one individual (P15) at day 0 and following the antibiotics at day 7, but by day 90 we found a complete BSAP gene (Figure 4A, Supplementary Figure S3A, and Supplementary Table S2). In another individual (P17), we found a new strain on day 7 that had an incomplete BSAP-3 gene, but on day 90 the strain was related to the pre-sample and also had a complete BSAP-3 gene. Finally, one individual of note, P4, had an incomplete BSAP-3 gene at days 0 and 7; however, the same *B. vulgatus* strain at day 90 had a complete BSAP-3 gene. This individual was the only example where we found a BSAP phenotype change from negative to positive without a strain change (Supplementary Figure S3A). The presence of the complete BSAP-3 gene in the dominant fecal strain of these two individuals that had incomplete genes at early times during the recovery is consistent with the function of the BSAP to specifically restrict *Bacteroides* spp. that are without complete BSAP genes.

We next analyzed the BSAP sequence changes using IGV [16]. For the BSAP-3 negative samples, we found eight samples in which the strains that were related at Day 0 and Day 90 had identical or few nucleotide differences in the 5’ and 3’ regions bracketing the BSAP-3 deletion (P2, P3, P9, P13, P18, P19, P20, and P21) supporting the recovery of the pre-antibiotic strain (Figure 4A and Figure S3A). For the samples without BSAP-2 genes, 10 samples that had no BSAP-2 genes for the Day 0 and Day 90 samples all had identical or few nucleotide differences in the 5′ and 3′ regions bracketing the deleted BSAP gene (P2, P9, P10, P11, P13, P15, P17, P18, P20, and P21) (Figure 4B and Figure S3B). Collectively, these results are consistent with the resiliency of the GIT strain reservoir to recover following perturbation.

**Figure 5:**
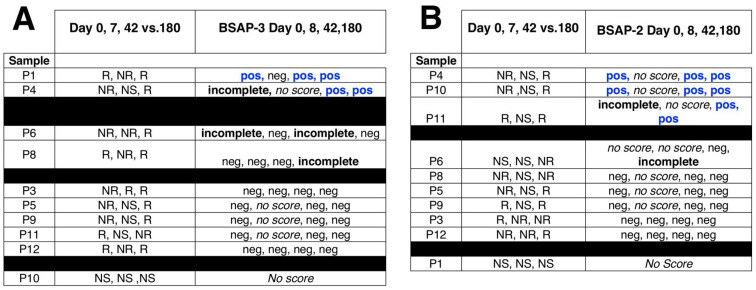
BSAP summary table for Palleja et al. data set. Gene analysis that includes WSS and BLAST was conducted for the Palleja et al. data set. For each sample, strain relatedness was determined by WSS analysis and the presence/absence of the (**A**) BSAP-3 and (**B**) BSAP-2 genes were determined by BLAST analysis. “R” indicates related strain, “NR” indicates not related strain observed, “NS” indicates no WSS score observed from WSS analysis. “pos” indicates BSAP-3/BSAP-2 gene was observed; “incomplete” indicates sequence fragment of BSAP-3/BSAP-2 gene was observed; “neg” indicates BSAP-3/BSAP-2 gene was not observed; “No score” indicates that no score was observed from WSS analysis.

To investigate the impact of a greater perturbation on the strain reservoir, we characterized the strain stability of individuals from the Palleja et al. data set that had been treated with a suppressive antibiotics cocktail consisting of three antibiotics (meropenem, gentamicin, and vancomycin) [7,19]. In this longitudinal study, we found that in some instances the treatment resulted in a strain change from the pre-antibiotic strain. Overall, 2 of 10 individuals showed complete BSAP-3 genes and 3 of 10 showed complete BSAP-2 genes (Figure 5, Supplementary Figure S4, and Supplementary Table S3). We found several examples where there was a change in an individual from an incomplete BSAP gene to a complete BSAP gene. Individual P1 had a strain change early after antibiotics that were BSAP-3 negative, but the same samples at later times (42 and 180 days) were the same strain as the pre-strain and both had a complete BSAP-3 gene (Figure 5A). Another individual (P4) that had an incomplete BSAP gene on day 0, had a strain change, and the new strains at day 42 and 180 both had complete BSAP-3 genes (Figure 5A). Similarly, in this individual, for *B. uniformis* there was also a new strain at days 42 and 180 that both had complete BSAP-2 genes (Figure 5A,B). Finally, for P11, there was no BSAP-2 change in the strain of *B. uniformis,* but they had a complete BSAP-2 gene (Figure 5B).

From IGV analysis for BSAP-3 in B. vulgatus, we found that only 1 of 10 samples (P1) had a complete BSAP-3 gene at days 0, 42, and 180 (Figure 5A). We did find two individuals, P11 and P12, where the *B. vulgatus* strain at Day 0 was related to the strain at Day 180. In the case of P11, the 5’ and 3’ regions at Day 0 and Day 180 showed that fecal dominant strains were different, whereas the 5’ and 3’ sequences from P12 were the same at Day 0 and 180 (Figure 5A and Figure S4A). For B uniformis, individuals P3 and P9 had related strains at Day 0 and 180 and both the 5’ and 3’ sequences were the same (Figure 5B and Figure S4B).

## 4. Discussion

In this study, we have analyzed the presence of genes encoding BSAP proteins in fecal *B. vulgatus* or *B, uniformis* strains from individuals with normal and perturbed GIT ecosystems. Our analysis combines the identification of BSAP genes (BSAP-2 and BSAP-3) with BLAST and the use of IGV tools to provide a comprehensive analysis of the fecal dominant *Bacteroides* spp. strains. Our studies support that the fecal dominant *Bacteroides* spp. strain is most probably maintained in a microbial strain reservoir within the GIT and, in some individuals, the selection of the dominant strain could be influenced by the BSAP.

Previous studies have shown the *Bacteroidales*-specific antimicrobial genes function in a species-specific fashion to restrict replication and subsequent colonization [13,29]. The analysis of the status of the BSAP genes then provides an opportunity to investigate possible interactions between *Bacteroides* spp. that occur in the GIT. For example, the presence of the BSAP genes would provide a selective advantage over the *Bacteroidales* microbe without BSAP in the GIT. To further define the presence or absence of the BSAP gene in the population, we first focused on the well-characterized HMP data set that contained paired samples from individuals taken at two different times, 3 months to 1 year apart [1,17]. Using WSS analysis, we had previously determined that, for the samples collected, the strains of *B. vulgatus* and *B. uniformis* of an individual were related to each other over this period [2]. Consistent with other studies, we found most individuals were either BSAP positive or negative [13,29]. From our examination of the longitudinal samples, we found that the BSAP phenotypes were stable over time in the unperturbed, normal GIT microbial ecosystem.

In most individuals, the *Bacteroides* spp. strain and BSAP gene were stable following treatment with single or multiple antibiotics. We did find specific instances where the BSAP changed during recovery from the antibiotic. For example, in one individual, P17 of the Raymond et al. study, we found that the cephalexin treatment resulted in the appearance of a new *B. vulgatus* strain on Day 7 where an incomplete BSAP-3 gene was replaced by the original *B. vulgatus* strain on day 90 that had a complete BSAP-3 gene. Individual P4 of the same Raymond et al. study had *B. vulgatus,* a strain with no BSAP-3 gene, at days 0 and 7, but had the *B. vulgatus* strain with a complete BSAP-3 gene at day 90. Similarly, for individual P1 of the *Palleja* et al. samples, the BSAP-3 changed from complete to incomplete from day 0 to day 7 post antibiotics. The Day 7 *B. vulgatus* strain was replaced by the pre-antibiotic BSAP-3 positive *B. vulgatus* strain at days 42 and 180. Collectively, these results support that in some individuals the complete BSAP gene in the GIT strain reservoir influenced the selection of the dominant fecal strain.

We used the IGV tool to visualize the BSAP genes and surrounding 5′ and 3′ regions. For those samples that were BSAP positive, all had complete BSAP genes although we did find that in some instances regions 5′ and 3′ had discrete deletions. In contrast, in IGV analysis of the samples with incomplete BSAP-3 and BSAP-2 genes, we found that for most of the samples the deletions encompassed the BSAP genes and surrounding 5′ and 3′ regions. Surprisingly, we found different individuals that had shared sequence deletions (Figure 2 and Figure 3). The sharing between different individuals suggests a common mechanism for the generation of deletions. One possibility would be that the deletions might be a result of a transposition event to delete the BSAP gene and surrounding DNA regions, although we did not find the consensus sequence for a known *Bacteroides* conjugative transposon (CTnDOT) [30,31]. Previous studies have reported that the *Bacteroides* genome region containing the BSAP gene was included in a region of integrative and conjugative elements which included numerous, yet undefined, putative transposases [32,33]. We also found that a few individuals with incomplete BSAP gene patterns were different between the sampling time points. Although the origin of these BSAP gene patterns is unknown, one possibility is that they occurred during a non-typical transposition process that left remnants of the BSAP gene. Environmental stress such as antibiotic treatment, nutritional changes, or even disease have been shown to enhance transposition [34,35,36]. Further understanding of the dynamics of the appearance of these deleted BSAP genes in an individual over time will be needed to determine if a correlation exists with microbial ecosystems that were or are currently under stress.

## 5. Conclusions

As discussed by Wexler and Goodman, the *Bacteroides* spp. are an ideal group in which to uncover fundamental principles for the persistence of microbes over time in the host [9]. The analysis of the BSAP gene following antibiotic treatment lends support for a possible role in the persistence and recovery of the dominant fecal strain of *Bacteroides* spp. Since the human microbiome is recognized as being diverse and generally individual specific, a strength of our study was the use of three longitudinal data sets that allowed the comparison of samples from the same individual pre and post treatment. Additional follow up studies with clinical implications would include an analysis of patients pre and post fecal transplant and those individuals that have had perturbation of their GIT ecosystem from oral drugs [37,38,39].

## Data Availability

The original sequencing data set of the stool samples used in this study was downloaded from the European Nucleotide Archive (accession numbers: PRJEB8094 for Raymond et al. [18], ERP022986 for Palleja et al. [19]) and https://portal.hmpdacc.org/, accessed on 13 June 2012 for the HMP data set.

## Supplementary Materials

The following supporting information can be downloaded at: https://www.mdpi.com/article/10.3390/life14050555/s1. Figure S1: IGV analysis for the HMP data set. Figure S2: IGV analysis for the control samples from Raymond et al. [18]. Figure S3: IGV analysis for the antibiotic treated samples from Raymond et al. [18]. Figure S4: IGV analysis for the antibiotic treated samples from Palleja et al. [19]. Table S1. Gene analysis summary table for the HMP data set. Table S2. Gene analysis summary table for the Raymond et al. [18]. Table S3. Gene analysis summary table for the Palleja et al. [19].
