# Supplementary material for: Bacteroidales-Specific Antimicrobial Genes Can Influence the Selection of the Dominant Fecal Strain of Bacteroides vulgatus and Bacteroides uniformis from the Gastrointestinal Tract Microbial Community
Source: Life (Basel). 2024 Apr 26;14(5):555. doi: 10.3390/life14050555 (PMC11121782; doi:10.3390/life14050555)
Supplement: Supplementary file 1 [file life-14-00555-s001.zip › Supplementary Figures.pdf]

Supplementary Figure S1A

A

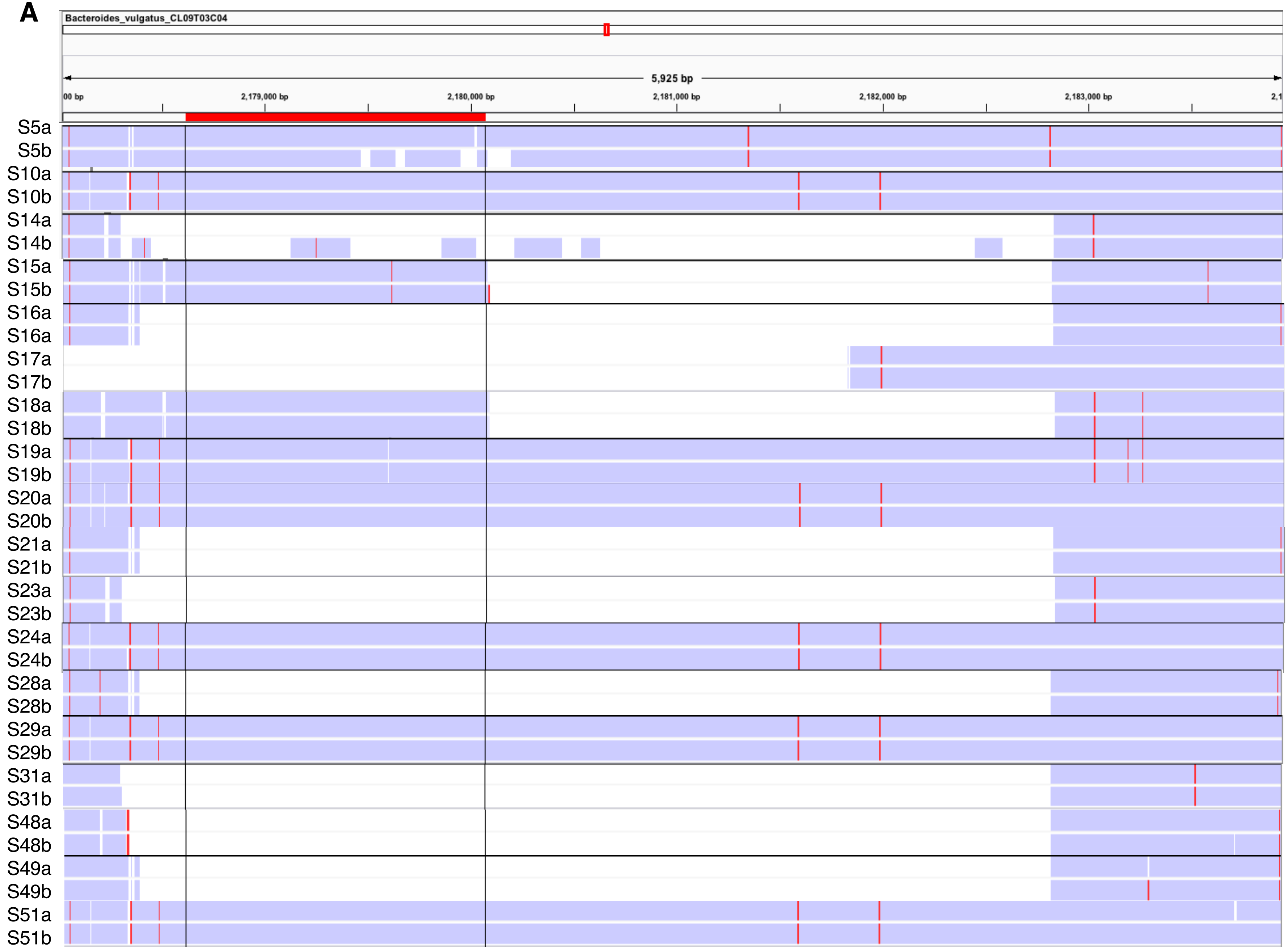

Supplementary Figure S1A Continued

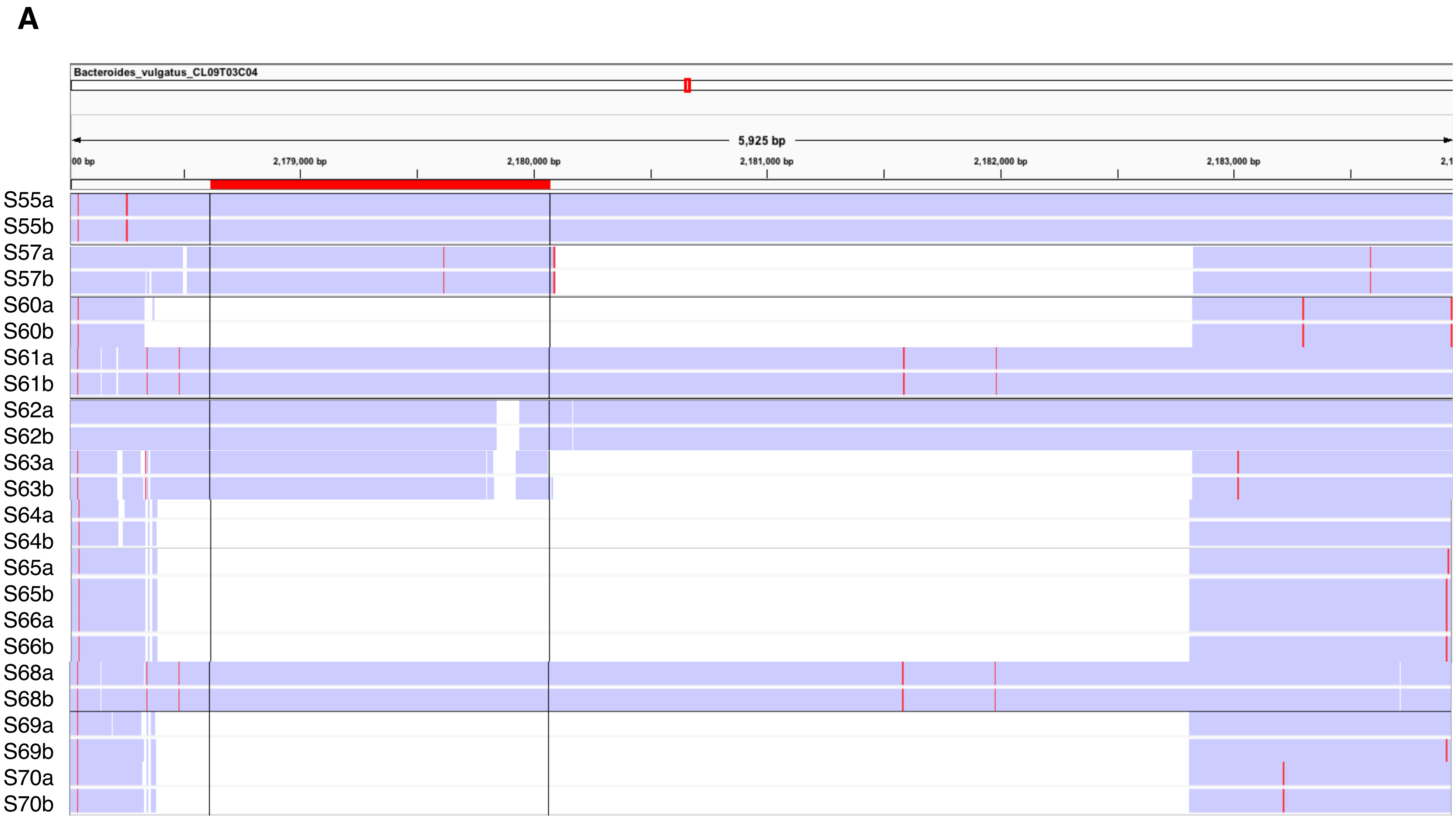

Supplementary Figure S1B

B

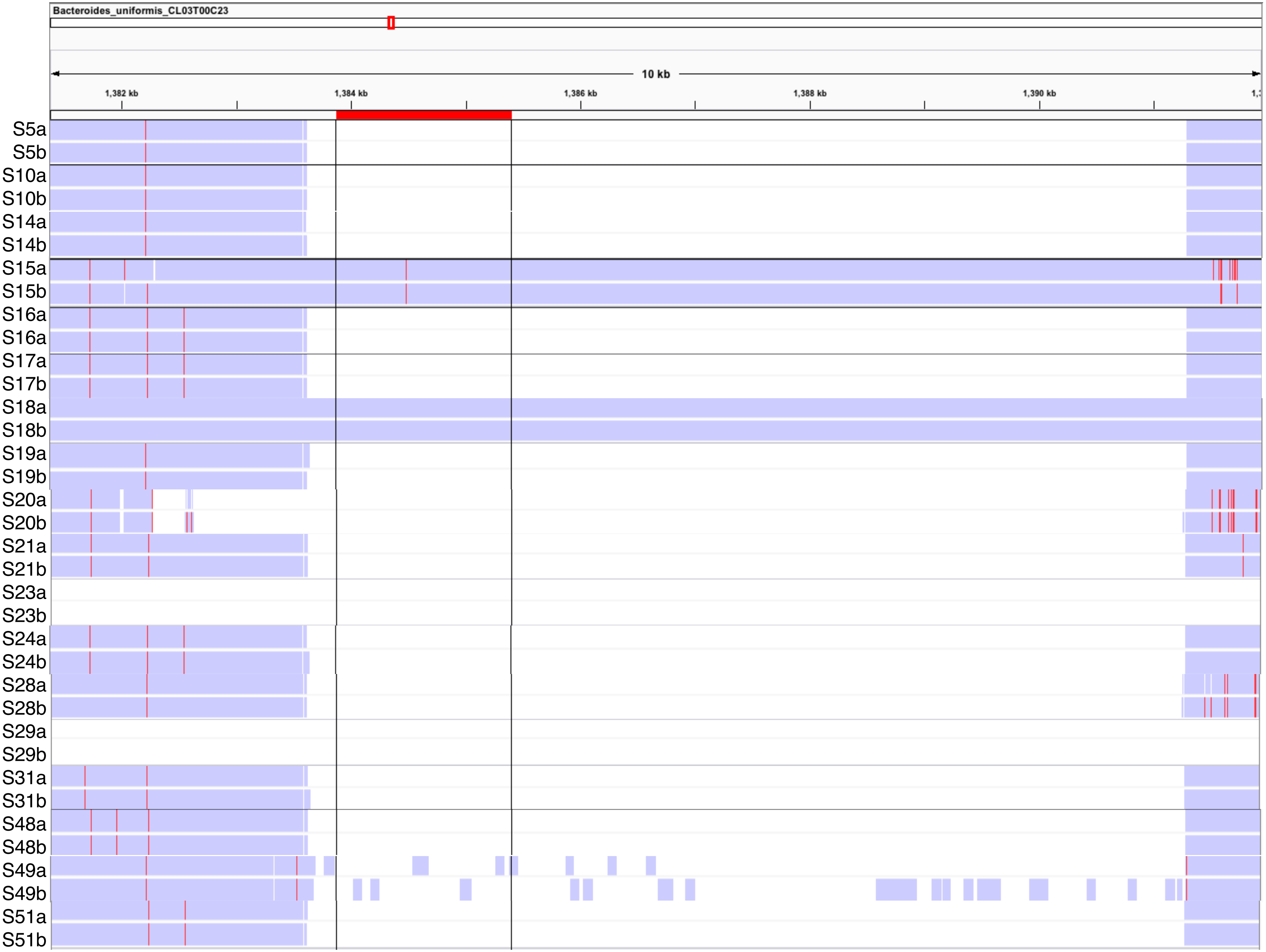

Supplementary Figure S1B Continued

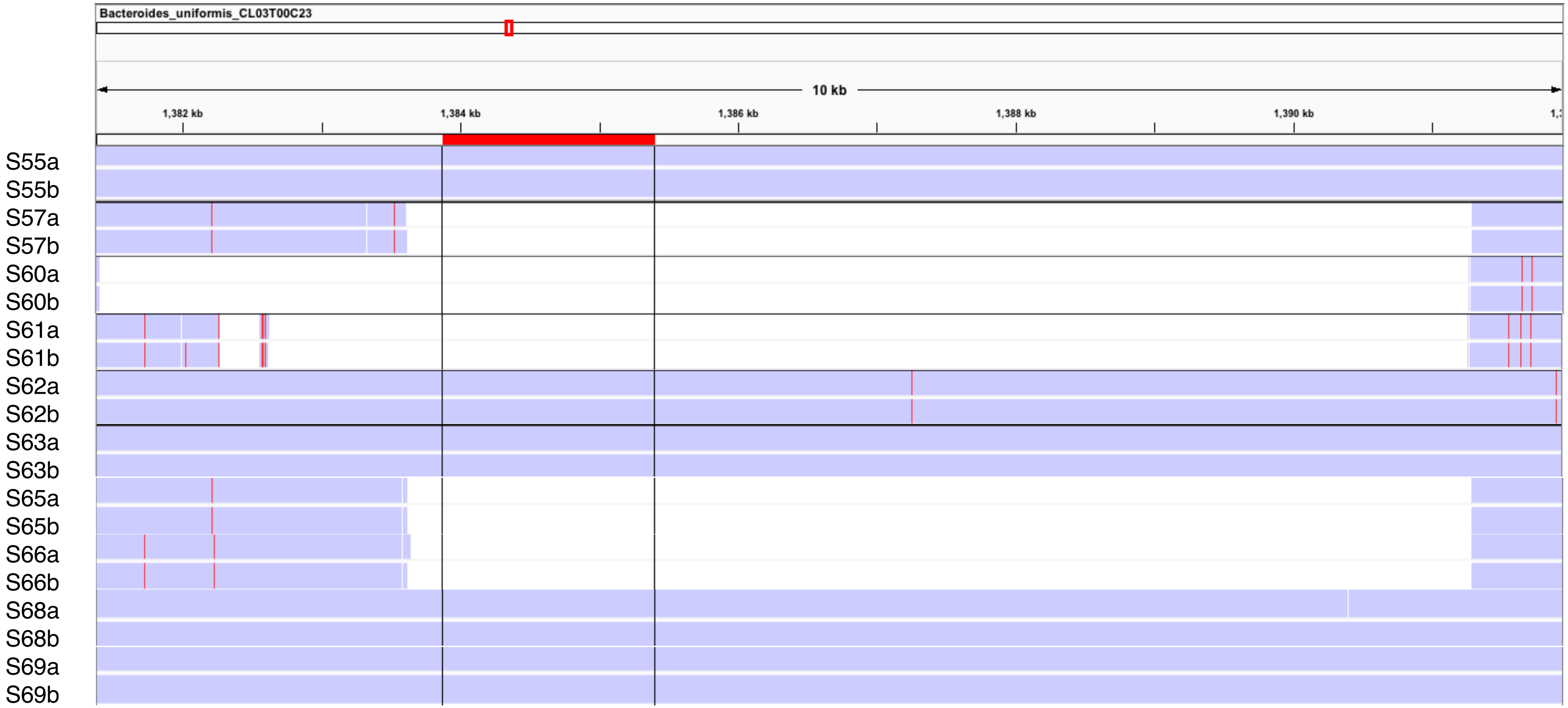

Supplementary Figure S1: IGV analysis for the HMP data set.

Aligned Bacteriocin genes, (A) BSAP-3 and (B) BSAP-2, were visualized using IGV for the HMP data set. For the BSAP-3, 2,178,016 to 2,183,973 regions of *B. vulgatus* were selected to display 5' and 3' sequences and BSAP-3 gene (red colored box: 2,178,616 to 2,180,073). For the BSAP-2, 1,381,372 to 1,392,000 regions of *B. uniformis* were selected to show 5' and 3' sequences and BSAP-2 gene (red colored box: 1,383,872-1,385,398).

Supplementary Figure S2A

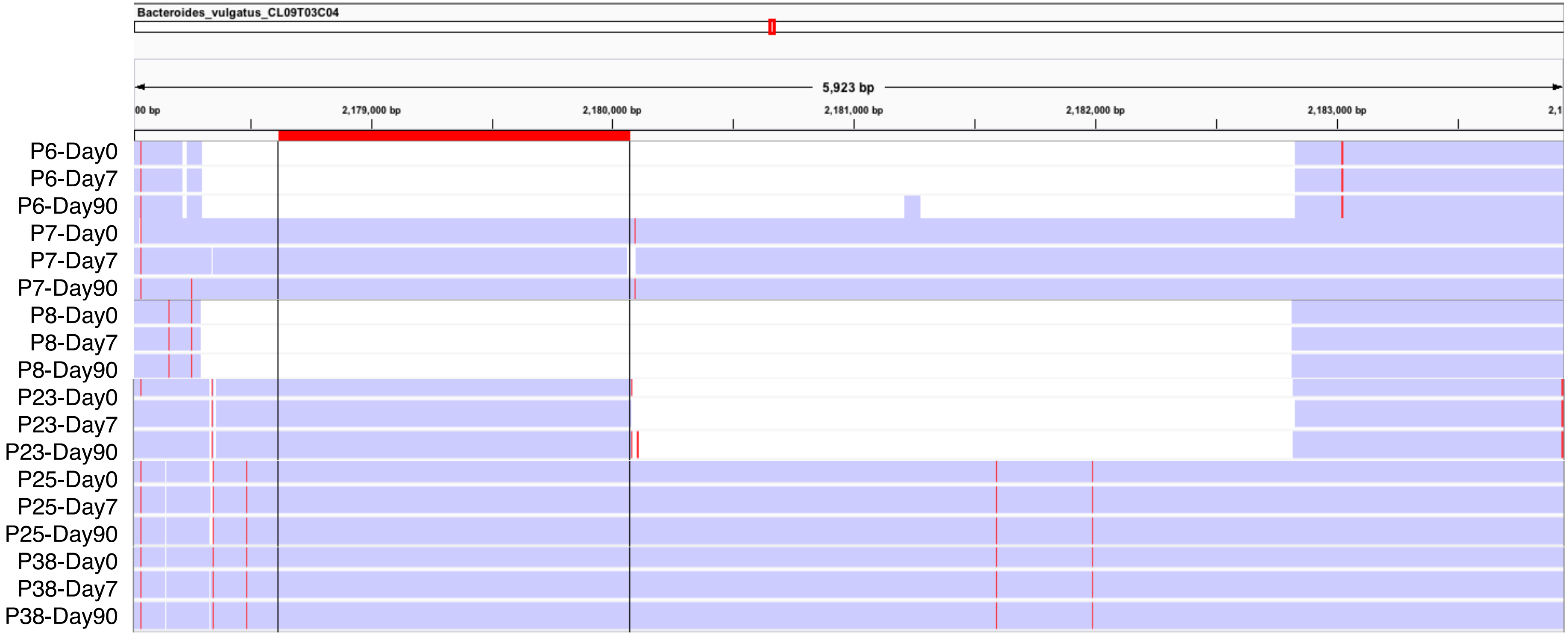

Supplementary Figure S2B

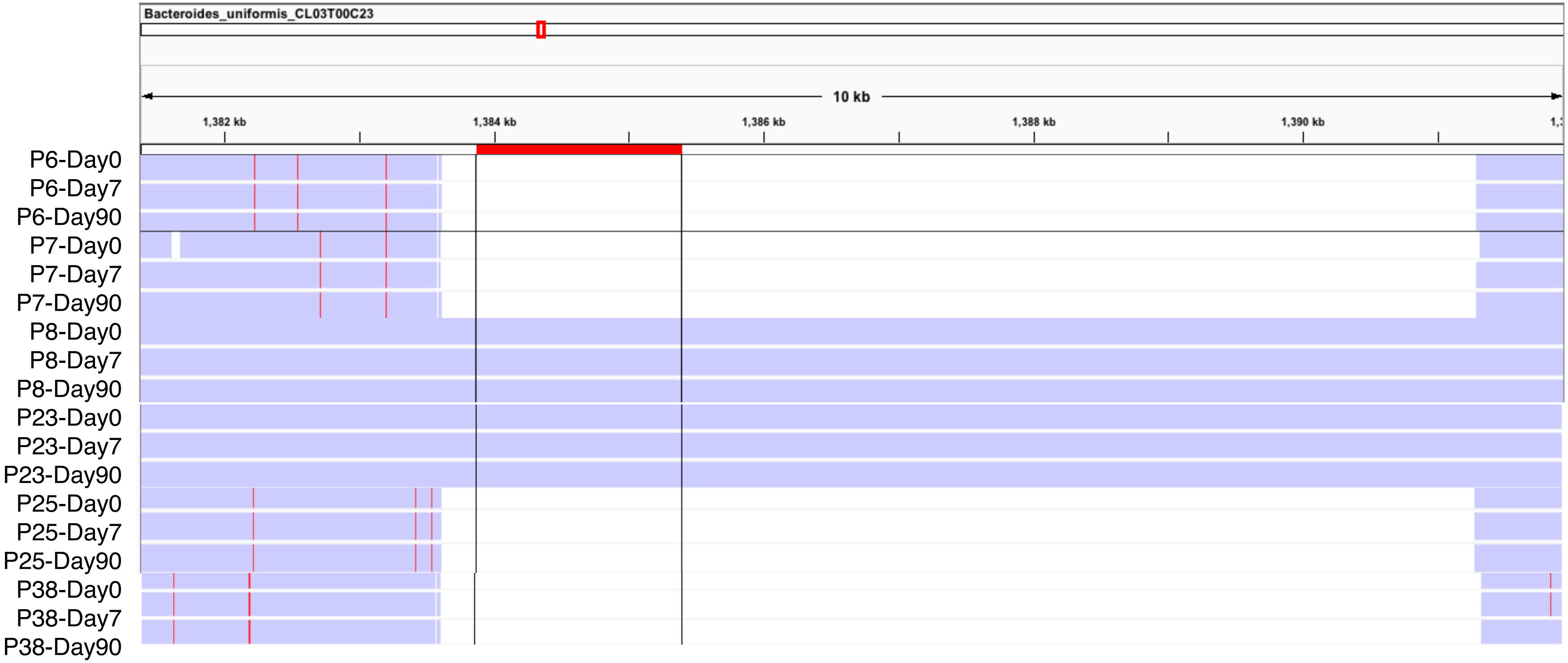

Supplementary Figure S2: IGV analysis for the control samples from Raymond et al. [18].
For control samples from Raymond et al., aligned Bacteriocin genes, (A) BSAP-3 and (B) BSAP-2, were visualized using IGV. For the BSAP-3, 2,178,016 to 2,183,973 regions of B. vulgatus were selected to display 5' and 3' sequences and BSAP-3 gene (red colored box: 2,178,616 to 2,180,073). For the BSAP-2, 1,381,372 to 1,392,000 regions of B. uniformis were selected to show 5' and 3' sequences and BSAP-2 gene (red colored box: 1,383,872-1,385,398).

Supplementary Figure S3A

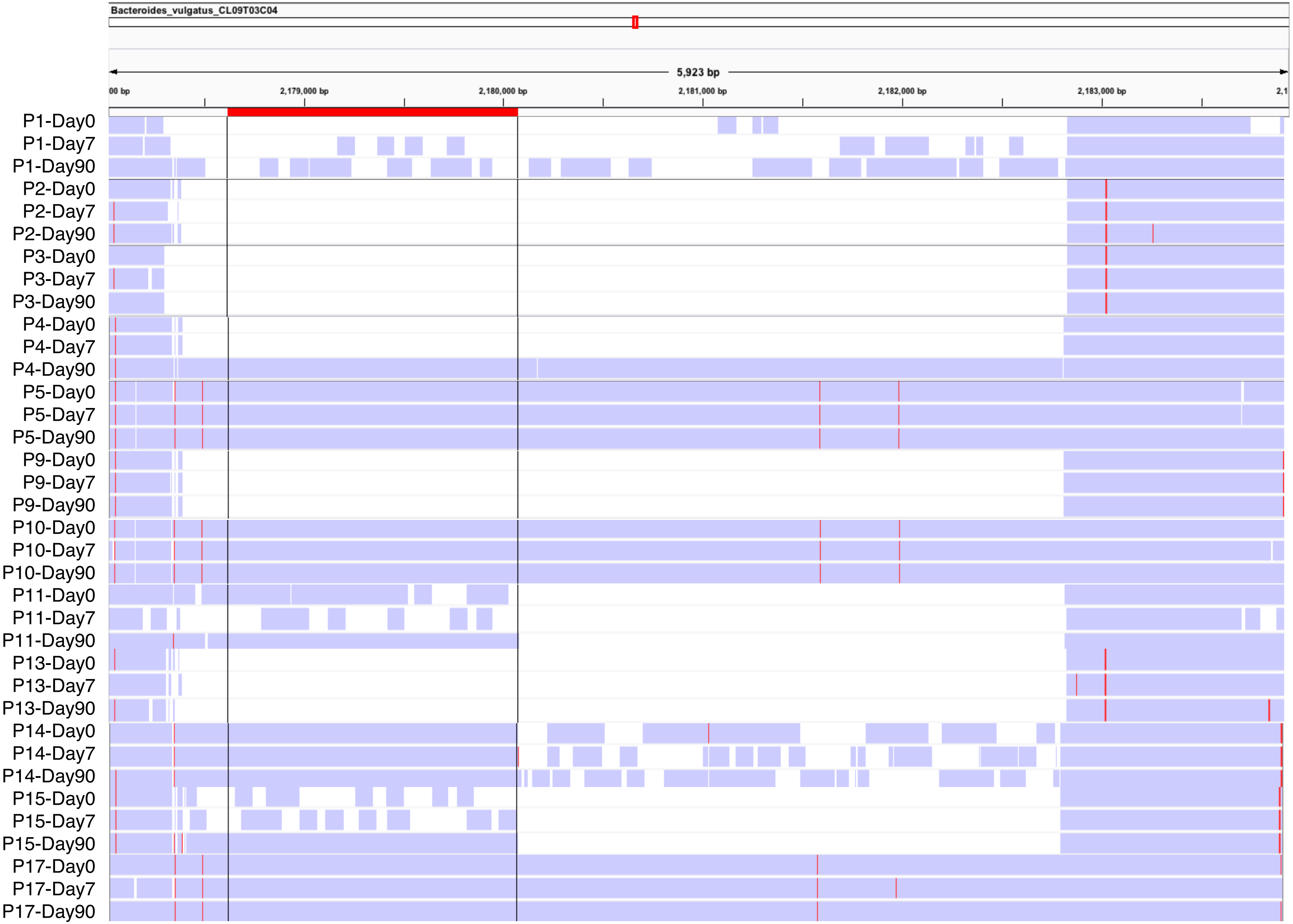

Supplementary Figure S3A - Continued

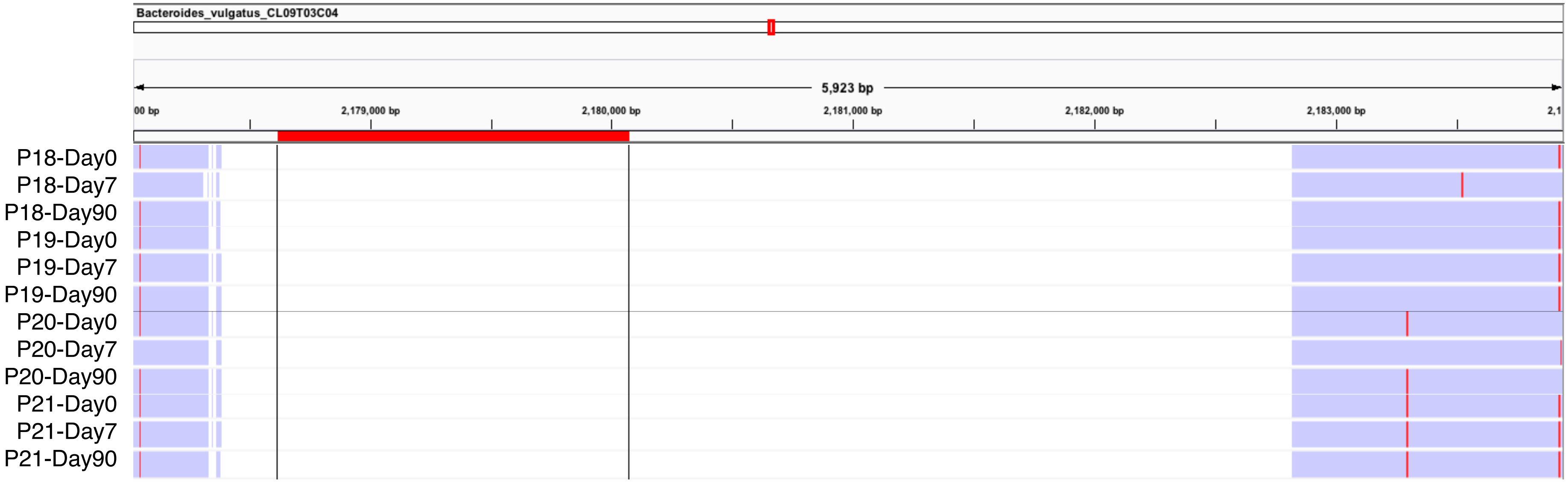

Supplementary Figure S3B

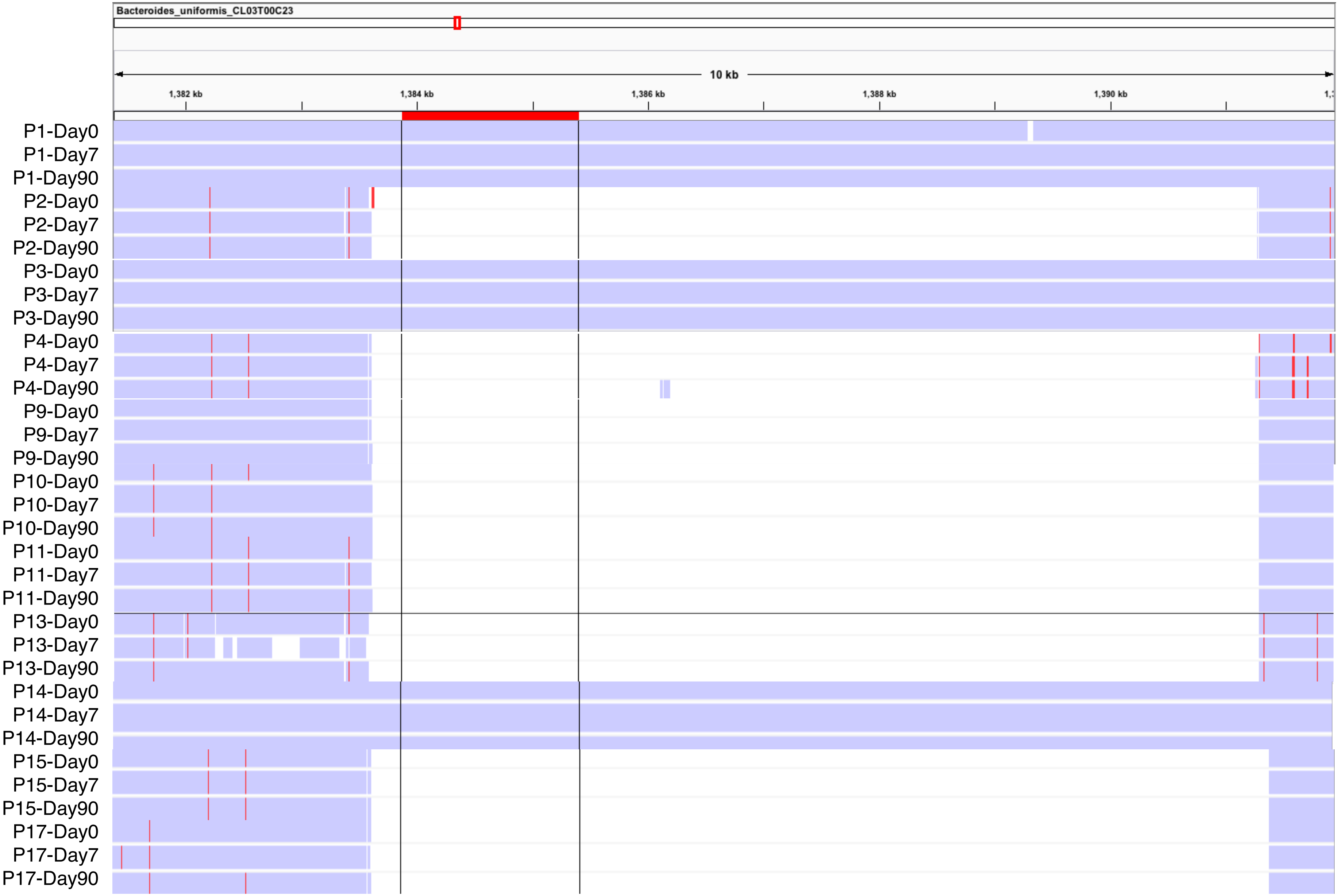

Supplementary Figure S3B - Continued

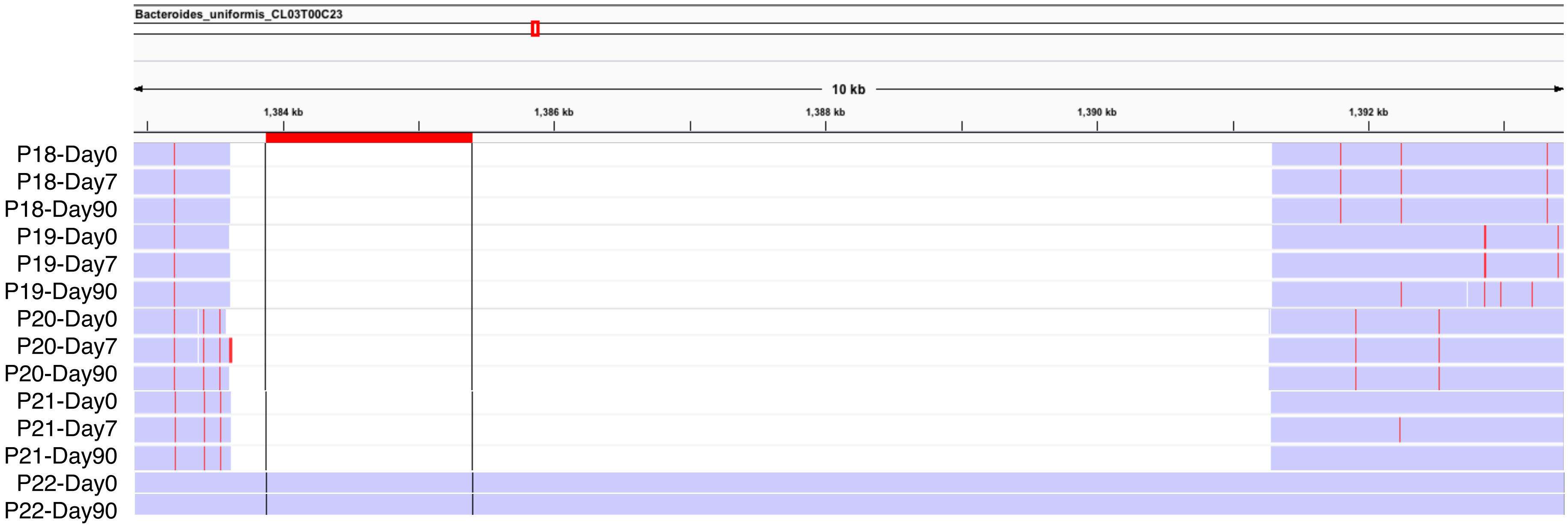

Supplementary Figure S3: IGV analysis for the antibiotic treated samples from Raymond et al. [18]. For antibiotic treated samples from Raymond et al., aligned Bacteriocin genes, (A) BSAP-3 and (B) BSAP-2, were visualized using IGV. For the BSAP-3, 2,178,016 to 2,183,973 regions of B. vulgatus were selected to display 5’ and 3’ sequences and BSAP-3 gene (red colored box: 2,178,616 to 2,180,073). For the BSAP-2, 1,381,372 to 1,392,000 regions of B. uniformis were selected to show 5’ and 3’ sequences and BSAP-2 gene (red colored box: 1,383,872-1,385,398).

Supplementary Figure S4A

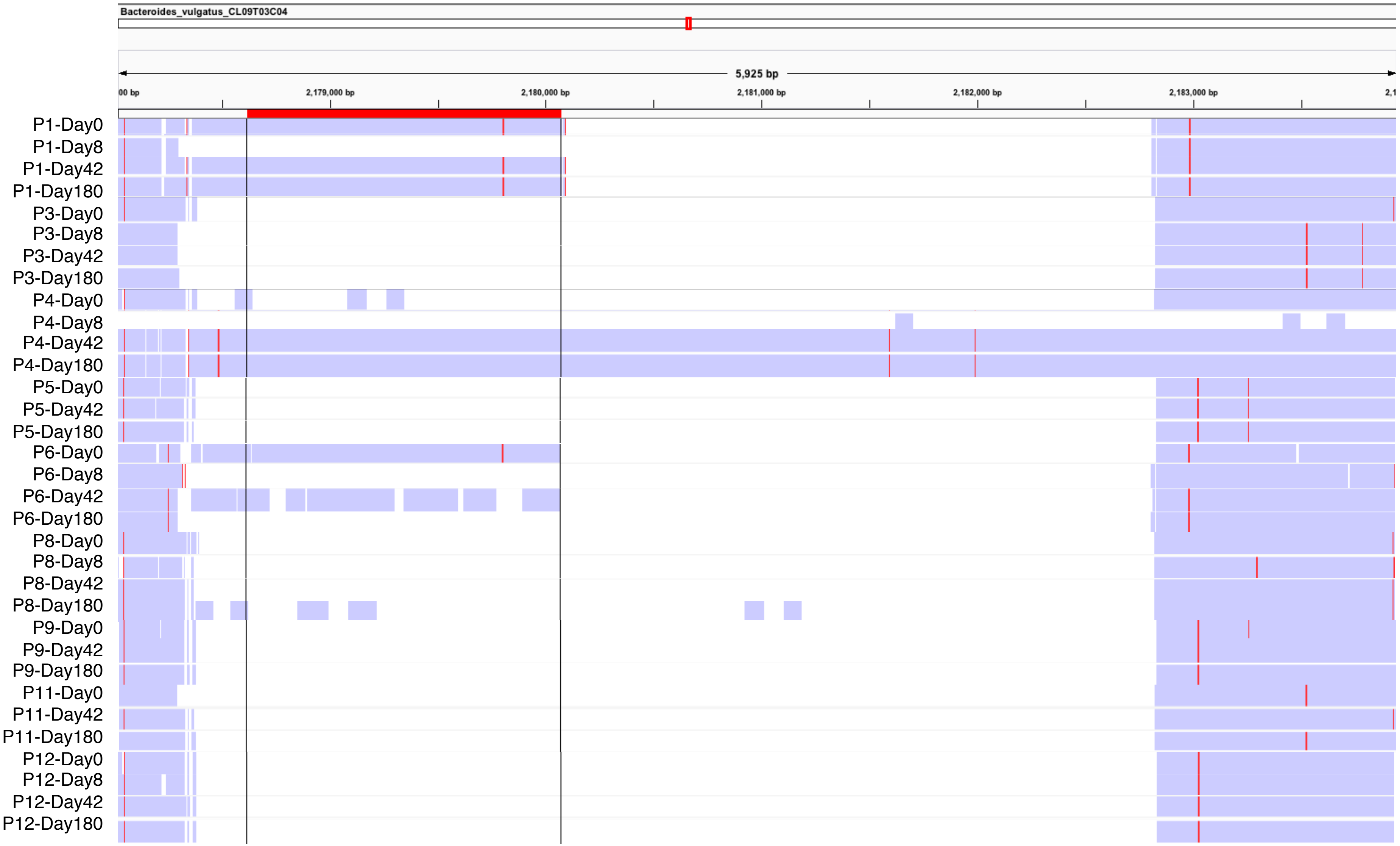

Supplementary Figure S4B

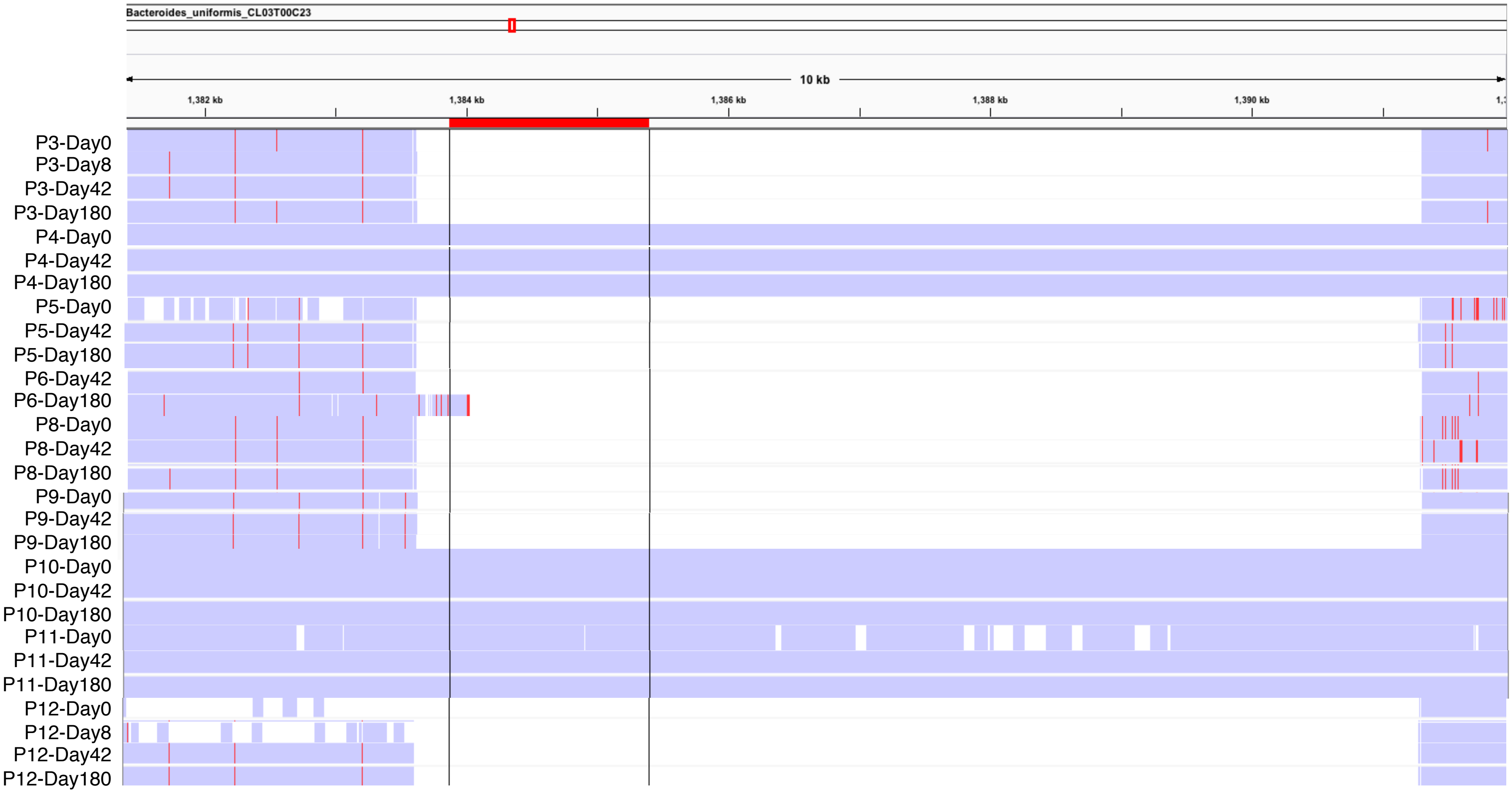

Supplementary Figure S4: IGV analysis for the antibiotic treated samples from Palleja et al. [19]. For antibiotic treated samples from Palleja et al., aligned Bacteriocin genes, (A) BSAP-3 and (B) BSAP-2, were visualized using IGV. For the BSAP-3, 2,178,016 to 2,183,973 regions of B. vulgatus were selected to display 5' and 3' sequences and BSAP-3 gene (red colored box: 2,178,616 to 2,180,073). For the BSAP-2, 1,381,372 to 1,392,000 regions of B. uniformis were selected to show 5' and 3' sequences and BSAP-2 gene (red colored box: 1,383,872-1,385,398).
