# Supplementary material for: Bacteroidales-Specific Antimicrobial Genes Can Influence the Selection of the Dominant Fecal Strain of Bacteroides vulgatus and Bacteroides uniformis from the Gastrointestinal Tract Microbial Community
Source: Life (Basel). 2024 Apr 26;14(5):555. doi: 10.3390/life14050555 (PMC11121782; doi:10.3390/life14050555)
Supplement: Supplementary file 1 [file life-14-00555-s001.zip › Supplementary Material.pdf]

Title: *Bacteroidales*-specific antimicrobial genes can influence the selection of the dominant fecal strain of *Bacteroides vulgatus* and *Bacteroides uniformis* from the gastrointestinal tract microbial community

Authors: Hyunmin Koo, Casey D. Morrow

Supplementary Material

For the WSS analysis, sequence reads from each sample were aligned to the 93 microbial reference genomes which were previously established based on the HMP dataset[1,2] using the Burrows-Wheeler aligner (BWA) tool[3]. Each sample was analyzed for multi-sample SNVs relative to the provided reference genome using the Genome Analysis Toolkit (GATK)[4]. The resulting multi-sample Variant Call Format (VCF) files were utilized for pairwise comparisons between all possible pairs of samples. This was done to determine the overall genome-wide SNV similarity for each microbial species. Samples with sequence coverage below 30% and sequence depth less than 3.5 against their given reference genome were excluded from the pairwise comparisons[1,5-8]. After quality-based filtering processes, *B. vulgatus* species that was able to provide the WSS score was selected from each data set. In order to identify related strains, the WSS score for the *B. vulgatus* species was compared to a previously established cut-off value from our ealier study. (For related strain pairs: WSS score>cut-off; for unrelated strain pairs: WSS score<cut-off)[1,9].

References

1. Kumar, R. et al. Identification of donor microbe species that colonize and persist long term in the recipient after fecal transplant for recurrent Clostridium difficile. NPJ biofilms and microbiomes 3, 1-4, doi:10.1038/s41522-017-0020-7 (2017).
2. Schloissnig, S. et al. Genomic variation landscape of the human gut microbiome. Nature 493, 45-50, doi:10.1038/nature11711 (2013).
3. Li, H. & Durbin, R. Fast and accurate long-read alignment with Burrows–Wheeler transform. Bioinformatics 26, 589-595, doi:10.1093/bioinformatics/btp698 (2010).
4. Van der Auwera, G. A. et al. From FastQ data to high confidence variant calls: the Genome Analysis Toolkit best practices pipeline. Curr Protoc Bioinformatics 43, 11.10.11-33, doi:10.1002/0471250953.bi1110s43 (2013).
5. Koo, H., Crossman, D. K. & Morrow, C. D. Strain Tracking to Identify Individualized Patterns of Microbial Strain Stability in the Developing Infant Gut Ecosystem. Frontiers in Pediatrics 8 (2020).
6. Koo, H. et al. Individualized recovery of gut microbial strains post antibiotics. NPJ Biofilms Microbiomes 5, 30, doi:10.1038/s41522-019-0103-8 (2019).
7. Koo, H., Hakim, J. A., Crossman, D. K., Lefkowitz, E. J. & Morrow, C. D. Sharing of gut microbial strains between selected individual sets of twins cohabitating for decades. PLOS One 14, e0226111, doi:10.1371/journal.pone.0226111 (2019).
8. Koo, H. & Morrow, C. D. Perturbation of the human gastrointestinal tract microbial ecosystem by oral drugs to treat chronic disease results in a spectrum of individual specific patterns of extinction and persistence of dominant microbial strains. PLOS One 15, e0242021, doi:10.1371/journal.pone.0242021 (2020).
9. Kumar, R. et al. New microbe genomic variants in patients fecal community following surgical disruption of the upper human gastrointestinal tract. Human Microbiome Journal 10, 37-42, doi:10.1016/j.humic.2018.10.002 (2018).
