# Supplementary material for: Bacteroidales-Specific Antimicrobial Genes Can Influence the Selection of the Dominant Fecal Strain of Bacteroides vulgatus and Bacteroides uniformis from the Gastrointestinal Tract Microbial Community
Source: Life (Basel). 2024 Apr 26;14(5):555. doi: 10.3390/life14050555 (PMC11121782; doi:10.3390/life14050555)
Supplement: Supplementary file 1 [file life-14-00555-s001.zip › Supplementary Tables.pdf]

**Supplementary Table S1. Gene analysis summary table for the HMP data set.**
Gene analysis which includes WSS and BLAST was conducted for the HMP data set. "+" indicates complete BSAP sequence was observed; "-" indicates no BSAP sequence was observed; "-" with yellow box indicates partial BSAP sequence was observed. Empty white box indicates not applicable due to a no WSS score or WSS score blow the cut-off value.

| | | | WSS score (*B. vulgatus*) | WSS score (*B. uniformis*) | *B. vulgatus* | *B. uniformis* |
|---|---|---|---|---|---|---|
| Dataset | Samples | | Cutoff: 95.1 | Cutoff: 97.8 | BSAP-3 | BSAP-2 |
| HMP | S10 | a | 100 | 100 | + | - |
| | | b | | | + | - |
| | S14 | a | 100 | 100 | - | - |
| | | b | | | - | - |
| | S15 | a | 100 | 99 | + | + |
| | | b | | | + | + |
| | S16 | a | 100 | 100 | - | - |
| | | b | | | - | - |
| | S17 | a | 100 | 100 | - | - |
| | | b | | | - | - |
| | S18 | a | 100 | 100 | - | + |
| | | b | | | - | + |
| | S19 | a | 99 | 100 | + | - |
| | | b | | | + | - |
| | S20 | a | 100 | 100 | + | - |
| | | b | | | + | - |
| | S21 | a | 99 | 100 | - | - |
| | | b | | | - | - |
| | S23 | a | 98 | 100 | - | - |
| | | b | | | - | - |
| | S24 | a | 100 | 100 | + | - |
| | | b | | | + | - |
| | S28 | a | 99 | 100 | - | - |
| | | b | | | - | - |
| | S29 | a | 100 | 100 | + | - |
| | | b | | | + | - |
| | S31 | a | 100 | 99 | - | - |
| | | b | | | - | - |
| | S48 | a | 100 | 100 | - | - |
| | | b | | | - | - |
| | S49 | a | 100 | 100 | - | - |
| | | b | | | - | - |

| | | | | | | |
|---|---|---|---|---|---|---|
| | S51 | a | 100 | 100 | + | - |
| | | b | | | + | - |
| | S55 | a | 100 | 100 | + | + |
| | | b | | | + | + |
| | S57 | a | 100 | 100 | + | - |
| | | b | | | + | - |
| | S5 | a | 99 | 100 | - | - |
| | | b | | | - | - |
| | S60 | a | 100 | 100 | - | - |
| | | b | | | - | - |
| | S61 | a | 100 | 100 | + | - |
| | | b | | | + | - |
| | S62 | a | 100 | 100 | - | + |
| | | b | | | - | + |
| | S63 | a | 99 | 100 | - | + |
| | | b | | | - | + |
| | S64 | a | 100 | 97 | - | NS |
| | | b | | | - | NS |
| | S65 | a | 100 | 100 | - | - |
| | | b | | | - | - |
| | S66 | a | 100 | 100 | - | - |
| | | b | | | - | - |
| | S68 | a | 100 | 100 | + | + |
| | | b | | | + | + |
| | S69 | a | 100 | 100 | - | + |
| | | b | | | - | + |
| | S70 | a | 100 | 97 | - | NS |
| | | b | | | - | NS |

**Supplementary Table S2. Gene analysis summary table for the Raymond et al. [18].** Gene analysis which includes WSS and BLAST was conducted for the Raymond et al. "+" indicates complete BSAP sequence was observed; "-" indicates no BSAP sequence was observed; "-" with yellow box indicates partial BSAP sequence was observed; "NS" indicates No WSS score observed. Empty white box indicates not applicable due to a no WSS score or WSS score blow the cut-off value.

| | | | WSS score (*B. vulgatus*) | WSS score (*B. uniformis*) | *B. vulgatus* | *B. uniformis* |
|---|---|---|---|---|---|---|
| Dataset | Samples | Days | Cutoff: 95.1 | Cutoff: 97.8 | BSAP-3 | BSAP-2 |
| Raymond et al. Antibiotics treated samples. | P1 | 0 | 94 | 89 | - | + |
| | | 7 | 96 | 91 | - | + |
| | | 90 | | | - | + |
| | P2 | 0 | 99 | 99 | - | - |
| | | 7 | 53 | 53 | - | - |
| | | 90 | | | - | - |
| | P3 | 0 | 100 | 100 | - | + |
| | | 7 | 34 | 96 | - | + |
| | | 90 | | | - | + |
| | P4 | 0 | 97 | 52 | - | - |
| | | 7 | 99 | 99 | - | - |
| | | 90 | | | + | - |
| | P5 | 0 | 100 | NS | + | NS |
| | | 7 | 100 | NS | + | NS |
| | | 90 | | | + | NS |
| | P9 | 0 | 100 | 100 | - | - |
| | | 7 | 100 | 100 | - | - |
| | | 90 | | | - | - |
| | P10 | 0 | 99 | 100 | + | - |
| | | 7 | 99 | 98 | + | - |
| | | 90 | | | + | - |
| | P11 | 0 | 100 | 100 | - | - |
| | | 7 | 89 | 36 | - | - |
| | | 90 | | | - | - |
| | P13 | 0 | 96 | 100 | - | - |
| | | 7 | 98 | 99 | - | - |
| | | 90 | | | - | - |
| | P14 | 0 | 100 | 99 | - | + |
| | | 7 | 99 | 100 | + | + |
| | | 90 | | | + | + |
| | P15 | 0 | 100 | 100 | - | - |
| | | 7 | 99 | 100 | - | - |
| | | 90 | | | + | - |
| | P17 | 0 | 100 | 100 | + | - |
| | | 7 | 95 | 95 | - | - |
| | | 90 | | | + | - |
| | P18 | 0 | 100 | 100 | - | - |
| | | 7 | 84 | 100 | - | - |

| | | | | | | |
|---|---|---|---|---|---|---|
| | | 90 | | | - | - |
| | P19 | 0 | 100 | 81 | - | - |
| | | 7 | 100 | 79 | - | - |
| | | 90 | | | - | - |
| | P20 | 0 | 100 | 98 | - | - |
| | | 7 | 99 | 100 | - | - |
| | | 90 | | | - | - |
| | P21 | 0 | 100 | 100 | - | - |
| | | 7 | 100 | 97 | - | - |
| | | 90 | | | - | - |
| | P22 | 0 | NS | 100 | NS | + |
| | | 7 | NS | NS | NS | NS |
| | | 90 | | | NS | + |
| Raymond et al. Control samples | P23 | 0 | 100 | 100 | + | + |
| | | 7 | 100 | 100 | + | + |
| | | 90 | | | + | + |
| | P25 | 0 | 99 | 100 | + | - |
| | | 7 | 99 | 100 | + | - |
| | | 90 | | | + | - |
| | P38 | 0 | 100 | 100 | + | - |
| | | 7 | 100 | 100 | + | - |
| | | 90 | | | + | - |
| | P6 | 0 | 100 | 100 | - | - |
| | | 7 | 100 | 100 | - | - |
| | | 90 | | | - | - |
| | P7 | 0 | 100 | 100 | - | - |
| | | 7 | 100 | 100 | - | - |
| | | 90 | | | + | - |
| | P8 | 0 | 100 | 100 | - | + |
| | | 7 | 100 | 100 | - | + |
| | | 90 | | | - | + |

**Supplementary Table S3. Gene analysis summary table for the Palleja et al. [19].**
Gene analysis which includes WSS and BLAST was conducted for the Palleja et al. "+" indicates complete BSAP sequence was observed; "-" indicates no BSAP sequence was observed; "-" with yellow box indicates partial BSAP sequence was observed; "NS" indicates No WSS score observed. Empty white box indicates not applicable due to a no WSS score or WSS score blow the cut-off value.

| | | | WSS score (*B. vulgatus*) | WSS score (*B. uniformis*) | *B. vulgatus* | *B. uniformis* |
|---|---|---|---|---|---|---|
| Dataset | Samples | Days | Cutoff: 95.1 | Cutoff: 97.8 | BSAP-3 | BSAP-2 |
| Palleja et al. | P1 | 0 | 100 | NS | + | NS |
| | | 8 | 89 | NS | - | NS |
| | | 42 | 100 | NS | + | NS |
| | | 180 | | | + | NS |
| | P3 | 0 | 2 | 99 | - | - |
| | | 8 | 100 | 20 | - | - |
| | | 42 | 100 | 93 | - | - |
| | | 180 | | | - | - |
| | P4 | 0 | 35 | 97 | - | + |
| | | 8 | NS | NS | NS | NS |
| | | 42 | 100 | 100 | + | + |
| | | 180 | | | + | + |
| | P5 | 0 | 73 | 14 | - | - |
| | | 8 | NS | NS | NS | NS |
| | | 42 | 100 | 100 | - | - |
| | | 180 | | | - | - |
| | P6 | 0 | 90 | NS | - | NS |
| | | 8 | 2 | NS | - | NS |
| | | 42 | 96 | 22 | - | - |
| | | 180 | | | - | - |
| | P8 | 0 | 100 | 13 | - | - |
| | | 8 | 19 | NS | - | NS |
| | | 42 | 100 | 19 | - | - |
| | | 180 | | | - | - |
| | P9 | 0 | 24 | 100 | - | - |
| | | 8 | NS | NS | NS | NS |
| | | 42 | 100 | 100 | - | - |
| | | 180 | | | - | - |
| | P10 | 0 | NS | 97 | NS | + |
| | | 8 | NS | NS | NS | NS |
| | | 42 | NS | 100 | NS | + |
| | | 180 | | | NS | + |

| | | | | | | |
|---|---|---|---|---|---|---|
| | P11 | 0 | 99 | 100 | - | - |
| | | 8 | NS | NS | NS | NS |
| | | 42 | 21 | 100 | - | + |
| | | 180 | | | - | + |
| | P12 | 0 | 100 | 13 | - | - |
| | | 8 | 83 | 10 | - | - |
| | | 42 | 100 | 100 | - | - |
| | | 180 | | | - | - |
